# Supplementary material for: Genetically Engineered Lung Cancer Cells for Analyzing Epithelial–Mesenchymal Transition
Source: Cells. 2019 Dec 15;8(12):1644. doi: 10.3390/cells8121644 (PMC6953058; doi:10.3390/cells8121644)
Supplement: Supplementary file 1 [file cells-08-01644-s001.pdf]

Figure S1. Alignment of the WT, expected and actual sequences encoding Vim (NM_003380) and the knock-in region. The expected KI and actual sequences matched perfectly while they differ to the WT in the expected regions. Two reads amplified using each of the following primers: 170F, 324R, 324F and 170R had been shown. Recoded bases corresponding to gRNA targeting site were coloured in red.

5'HA; VIM missing part; T2A; mCardinal; 3xFLAG; 3'HA; VIM gRNA target site

```
expected    TCGAACCCAAGTACACTCTTGCATTCTATGCTTTAAGTTAAATGCAAACTCCTTTTTCCT 60
genomic     --------------------------------------TAAATGCAAACTCCTTTTTCCT 60
170FI       ------------------------------------------------------------ 60
170FII      ------------------------------------------------------------ 60
324RI       ------------------------------------------------------------ 60
324RII      ------------------------------------------------------------ 60
324FI       ------------------------------------------------------------ 60
324FII      ------------------------------------------------------------ 60
170RI       ------------------------------------------------------------ 60
170RII      ------------------------------------------------------------ 60

expected    TCTTCCTGCTGCAAGTACTATCTCATCCTGATGCTCAAGAGTGTCAGGGCCTGGGTTTCC 120
genomic     TCTTCCTGCTGCAAGTACTATCTCATCCTGATGCTCAAGAGTGTCAGGGCCTGGGTTTCC 120
170FI       --------------------TCTCATCCTGATGCTCAAGAGTGTCAGGGCCTGGGTTTCC 120
170FII      ---------------------CTCATCCTGATGCTCAAGAGTGTCAGGGCCTGGGTTTCC 120
324RI       ------------------------------------------------------------ 120
324RII      ------------------------------------------------------------ 120
324FI       ------------------------------------------------------------ 120
324FII      ------------------------------------------------------------ 120
170RI       ------------------------------------------------------------ 120
170RII      ------------------------------------------------------------ 120

expected    AAACAGAGACTACCCTAAAATTATTTGGCGAGTAGTACTTTACACAATTGCCTCTCCCCC 180
genomic     AAACAGAGACTACCCTAAAATTATTTGGCGAGTAGTACTTTACACAATTGCCTCTCCCCC 180
170FI       AAACAGAGACTACCCTAAAATTATTTGGGGAGTAGTACTTTACACAATTGCCTCTCCCCC 180
170FII      AAACAGAGACTACCCTAAAATTATTTGGGGAGTAGTACTTTACACAATTGCCTCTCCCCC 180
324RI       ------------------------------------------------------------ 180
324RII      ------------------------------------------------------------ 180
324FI       ------------------------------------------------------------ 180
324FII      ------------------------------------------------------------ 180
170RI       ------------------------------------------------------------ 180
170RII      ------------------------------------------------------------ 180

expected    ACAAATCATAATTGTTTCAGTAAAATGGTTACTTGGTTTTTCCAAGAAAAAACTCGTTTT 240
genomic     ACAAATCATAATTGTTTCAGTAAAATGGTTACTTGGTTTTTCCAAGAAAAAACTCGTTTT 240
170FI       ACAAATCATAATTGTTTCAGTAAAATGGTTACTTGGTTTTTCCAAGAAAAAACTCGTTTT 240
170FII      ACAAATCATAATTGTTTCAGTAAAATGGTTACTTGGTTTTTCCAAGAAAAAACTCGTTTT 240
324RI       ------------------------------------------------------------ 240
324RII      ------------------------------------------------------------ 240
324FI       ------------------------------------------------------------ 240
324FII      ------------------------------------------------------------ 240
```

```
170RI       ------------------------------------------------------------ 240
170RII      ------------------------------------------------------------ 240

expected    TACTCATTTTTGGCCTGTTTGTTTATTTAGAAACTAATCTGGATTCACTCCCTCTGGTTG 300
genomic     TACTCATTTTTGGCCTGTTTGTTTATTTAGAAACTAATCTGGATTCACTCCCTCTGGTTG 300
170FI       TACTCATTTTTGGCCTGTTTGTTTATTTAGAAACTAATCTGGATTCACTCCCTCTGGTTG 300
170FII      TACTCATTTTTGGCCTGTTTGTTTATTTAGAAACTAATCTGGATTCACTCCCTCTGGTTG 300
324RI       ------------------------------------------------------------ 300
324RII      ------------------------------------------------------------ 300
324FI       ------------------------------------------------------------ 300
324FII      ------------------------------------------------------------ 300
170RI       ------------------------------------------------------------ 300
170RII      ------------------------------------------------------------ 300

expected    ATACCCACTCAAAAAGGACACTTCTGATTAAGACGGTTGAAACTAGAGATGGACAGGTTG 360
genomic     ATACCCACTCAAAAAGGACACTTCTGATTAAGACGGTTGAAACTAGAGATGGACAGGTTG 360
170FI       ATACCCACTCAAAAAGGACACTTCTGATTAAGACGGTTGAAACTAGAGATGGACAGGTTG 360
170FII      ATACCCACTCAAAAAGGACACTTCTGATTAAGACGGTTGAAACTAGAGATGGACAGGTTG 360
324RI       ------------------------------------------------------------ 360
324RII      ------------------------------------------------------------ 360
324FI       ------------------------------------------------------------ 360
324FII      ------------------------------------------------------------ 360
170RI       ------------------------------------------------------------ 360
170RII      ------------------------------------------------------------ 360

expected    GTATCTTTTAAGGAAAAAAATAGGGTAATCTCAGACAGGAGTTGATATATTTTAAAATCAG 420
genomic     GTATCTTTTAAGGAAAAAAATAGGGTAATCTCAGACAGGAGTTGATATATTTTAAAATCAG 420
170FI       GTATCTTTTAAGGAAAAAAATAGGGTAATCTCAGACAGGAGTTGATATATTTTAAAATCAG 420
170FII      GTATCTTTTAAGGAAAAAAATAGGGTAATCTCAGACAGGAGTTGATATATTTTAAAATCAG 420
324RI       ----------------------------------------------------AAAATCAG 420
324RII      ----------------------------------------------------AAAATCAG 420
324FI       ------------------------------------------------------------ 420
324FII      ------------------------------------------------------------ 420
170RI       ------------------------------------------------------------ 420
170RII      ------------------------------------------------------------ 420

expected    TGAATCTGAATCTCAGATACAGCTGGCTAATTTGAGAGGTTCAGGTTTCATTCATGCCTA 480
genomic     TGAATCTGAATCTCAGATACAGCTGGCTAATTTGAGAGGTTCAGGTTTCATTCATGCCTA 480
170FI       TGAATCTGAATCTCAGATACAGCTGGCTAATTTGAGAGGTTCAGGTTTCATTCATGCCTA 480
170FII      TGAATCTGAATCTCAGATACAGCTGGCTAATTTGAGAGGTTCAGGTTTCATTCATGCCTA 480
324RI       TGAATCTGAATCTCAGATACAGCTGGCTAATTTGAGAGGTTCAGGTTTCATTCATGCCTA 480
324RII      TGAATCTGAATTTCAGATACAGCTGGCTAATTTGAGAGGTTCAGGTTTCATTCATGCCTA 480
324FI       ------------------------------------------------------------ 480
324FII      ------------------------------------------------------------ 480
170RI       ------------------------------------------------------------ 480
```

```
170RII      ------------------------------------------------------------ 480

expected    CTAAAAAAAGAATAGGC-TTCTTCTTCCAGCAGTACACACAGCCAACTAATTATTTGGCT 540
genomic     CTAAAAAAAGAATAGGC-TTCTTCTTCCAGCAGTACACACAGCCAACTAATTATTTGGCT 540
170FI       CTAAAAAAAGAATAGGC-TTCTTCTTCCAGCAGTACACACAGCCAACTAATTATTTGGCT 540
170FII      CTAAAAAAAGAATAGGC-TTCTTCTTCCAGCAGTACACACAGCCAACTAATTATTTGGCT 540
324RI       CTAAAAAAAGAATAGGCTTTTTTTTTCCAGCAGTACACACAGCCAACTAATTATTTGGCT 540
324RII      CTAAAAAAAGAATAGGC-TTCTTTTTCCAGCAGTACACACAGCCAACTAATTATTTGGCT 540
324FI       ------------------------------------------------------------ 540
324FII      ------------------------------------------------------------ 540
170RI       ------------------------------------------------------------ 540
170RII      ------------------------------------------------------------ 540

expected    CCTGGATGTGAAGTTGAGATAGCAGTCTTCCTGTGCTCCAGAATTAGTGATTTGCTTTGG 600
genomic     CCTGGATGTGAAGTTGAGATAGCAGTCTTCCTGTGCTCCAGAATTAGTGATTTGCTTTGG 600
170FI       CCTGGATGTGAAGTTGAGATAGCAGTCTTCCTGTGCTCCAGAATTAGTGATTTGCTTTGG 600
170FII      CCTGGATGTGAAGTTGAGATAGCAGTCTTCCTGTGCTCCAGAATTAGTGATTTGCTTTGG 600
324RI       CCTGGATGTGAAGTTGAGATAGCAGTCTTCCTGTGCTCCAGAATTAGTGATTTGCTTTGG 600
324RII      CCTGGATGTGAAGTTGAGATAGCAGTCTTCCTGTGCTCCAGAATTAGTGATTTGCTTTGG 600
324FI       ------------------------------------------------------------ 600
324FII      ------------------------------------------------------------ 600
170RI       ------------------------------------------------------------ 600
170RII      ------------------------------------------------------------ 600

expected    TGCTTAATTTGAAGTGGGAGTAAGCTTCCTTAAACCACTTCCTAAAGCAGCTACATGAAA 660
genomic     TGCTTAATTTGAAGTGGGAGTAAGCTTCCTTAAACCACTTCCTAAAGCAGCTACATGAAA 660
170FI       TGCTTAATTTGAAGTGGGAGTAAGCTTCCTTAAACCACTTCCTAAAGCAGCTACATGAAA 660
170FII      TGCTTAATTTGAAGTGGGAGTAAGCTTCCTTAAACCACTTCCTAAAGCAGCTACATGAAA 660
324RI       TGCTTAATTTGAAGTGGGAGTAAGCTTCCTTAAACCACTTCCTAAAGCAGCTACATGAAA 660
324RII      TGCTTAATTTGAAGTGGGAGTAAGCTTCCTTAAACCACTTCCTAAAGCAGCTACATGAAA 660
324FI       ------------------------------------------------------------ 660
324FII      ------------------------------------------------------------ 660
170RI       ------------------------------------------------------------ 660
170RII      ------------------------------------------------------------ 660

expected    CAGCTTCACTAGACTACCTCAATATGAGGAATGTTTTGATCCTGGACATATGGTGTCTTC 720
genomic     CAGCTTCACTAGACTACCTCAATATGAGGAATGTTTTGATCCTGGACATATGGTGTCTTC 720
170FI       CAGCTTCACTAGACTACCTCAATATGAGGAATGTTTTGATCCTGGACATATGGTGTCTTC 720
170FII      CAGCTTCACTAGACTACCTCAATATGAGGAATGTTTTGATCCTGGACATATGGTGTCTTC 720
324RI       CAGCTTCACTAGACTACCTCAATATGAGGAATGTTTTGATCCTGGACATATGGTGTCTTC 720
324RII      CAGCTTCACTAGACTACCTCAATATGAGGAATGTTTTGATCCTGGACATATGGTGTCTTC 720
324FI       ------------------------------------------------------------ 720
324FII      ------------------------------------------------------------ 720
170RI       ------------------------------------------------------------ 720
170RII      ------------------------------------------------------------ 720
```

```
expected   CTACCTCCATACTTTATAGATTCCTAAACCCATCTATATAATACAAGCATGTGCCATACG 780
genomic    CTACCTCCATACTTTATAGATTCCTAAACCCATCTATATAATACAAGCATGTGCCATACG 780
170FI      CTACCTCCATACTTTATAGATTCCTAAACCCATCTATATAATACAAGCATGTGCCATACG 780
170FII     CTACCTCCATACTTTATAGATTCCTAAACCCATCTATATAATACAAGCATGTGCCATACG 780
324RI      CTACCTCCATACTTTATAGATTCCTAAACCCATCTATATAATACAAGCATGTGCCATACG 780
324RII     CTACCTCCATACTTTATAGATTCCTAAACCCATCTATATAATACAAGCATGTGCCATACG 780
324FI      ------------------------------------------------------------ 780
324FII     ------------------------------------------------------------ 780
170RI      ------------------------------------------------------------ 780
170RII     ------------------------------------------------------------ 780

expected   ATCATTTAGTTTCTTATTACCTCCCTATGCCAGGAAAGAAATAGTTGCAATTTATTGTAG 840
genomic    ATCATTTAGTTTCTTATTACCTCCCTATGCCAGGAAAGAAATAGTTGCAATTTATTGTAG 840
170FI      ATCATTTAGTTTCTTATTACCTCCCTATGCCAGGAAAGAAATAGTTGCAATTTATTGTAG 840
170FII     ATCATTTAGTTTCTTATTACCTCCCTATGCCAGGAAAGAAATAGTTGCAATTTATTGTAG 840
324RI      ATCATTTAGTTTCTTATTACCTCCCTATGCCAGGAAAGAAATAGTTGCAATTTATTGTAG 840
324RII     ATCATTTAGTTTCTTATTACCTCCCTATGCCAGGAAAGAAATAGTTGCAATTTATTGTAG 840
324FI      ------------------------------------------------------------ 840
324FII     ------------------------------------------------------------ 840
170RI      ------------------------------------------------------------ 840
170RII     ------------------------------------------------------------ 840

expected   TCATCATGAAATCTTCCCTTGCACATAAATTTAAAATGTACCTGCTGCACATTTTAATAT 900
genomic    TCATCATGAAATCTTCCCTTGCACATAAATTTAAAATGTACCTGCTGCACATTTTAATAT 900
170FI      TCATCATGAAATCTTCCCTTGCACATAAATTTAAAATGTACCTGCTGCACATTTTAATAT 900
170FII     TCATCATGAAATCTTCCCTTGCACATAAATTTAAAATGTACCTGCTGCACATTTTAATAT 900
324RI      TCATCATGAAATCTTCCCTTGCACATAAATTTAAAATGTACCTGCTGCACATTTTAATAT 900
324RII     TCATCATGAAATCTTCCCTTGCACATAAATTTAAAATGTACCTGCTGCACATTTTAATAT 900
324FI      ------------------------------------------------------------ 900
324FII     ------------------------------------------------------------ 900
170RI      ------------------------------------------------------------ 900
170RII     ------------------------------------------------------------ 900

expected   GTCTTAATTGCTTTTAAACTTGGCTGTATTGTGTACAACTATTATACCATCTTTTATAAA 960
genomic    GTCTTAATTGCTTTTAAACTTGGCTGTATTGTGTACAACTATTATACCATCTTTTATAAA 960
170FI      GTCTTAATTGCTTTTAAACTTGGCTGTATTGTGTACAACTATTATACCATCTTTTATAAA 960
170FII     GTCTTAATTGCTTTTAAACTTGGCTGTATTGTGTACAACTATTATACCATCTTTTATAAA 960
324RI      GTTTTAATTGCTTTTAAACTTGGCTGTATTGTGTACAACTATTATACCATCTTTTATAAA 960
324RII     GTTTTAATTGCTTTTAAACTTGGCTGTATTGTGTACAACTATTATACCATCTTTTATAAA 960
324FI      ------------------------------------------------------------ 960
324FII     ------------------------------------------------------------ 960
170RI      ------------------------------------------------------------ 960
170RII     ------------------------------------------------------------ 960
```

```
expected   CACAGTTTTTTAAGAAATTTCTTTTTGTAAGTTACAACATTCCACTGGAtccttatAGGA 1020
genomic    CACAGTTTTTTAAGAAATTTCTTTTTGTAAGTTACAACATTCCACTGGATCCTTATATTG 1020
170FI      CACAGTTTTTTAAGAAATTTCTTTTTGTAAGTTACAACATTCCACTGGATCCTTATAGGA 1020
170FII     CACAGTTTTTTAAGAAATTTCTTTTTGTAAGTTACAACATTCCACTGGATCCTTATAGGA 1020
324RI      CMCAGTTTTTTWAGAAATTTCTTTTTGTAAGTTACAACMTTYCACTGGATCCTTATWGGA 1020
324RII     CACAGTTTTTTWAGAAATTTCTTTTTGTAAGTTACAACMTTYCACYGGATCCTTATWGGA 1020
324FI      ------------------------------------------------------------ 1020
324FII     ------------------------------------------------------------ 1020
170RI      ------------------------------------------------------------ 1020
170RII     ------------------------------------------------------------ 1020

expected   CATATAATACAAGAGGGTCTTGTGTGTCTGCCCCTTCTAGTTTTCACTCATGCAGAAGCA 1080
genomic    CCT--------------------------------------------------------- 1080
170FI      CATATAATACAAGAGGGTCTTGTGTGTCTGCCCCTTCTAGTTTTCACTCATGCAGAAGCA 1080
170FII     CATATAATACAAGAGGGTCTTGTGTGTCTGCCCCTTCTAGTTTTCACTCATGCAGAAGCA 1080
324RI      CATATAATACMAGAGGGTCTTGKGKGTCTGCCCCTTCTAGTTTTCAMTCATGCAGAAGCA 1080
324RII     CATATAATACMAGAGGGTCTTGKGKGTCTGCCCCTTCTAGTTTTCAMTCATGCAGAAGCA 1080
324FI      ------------------------------------------------------------ 1080
324FII     ------------------------------------------------------------ 1080
170RI      ------------------------------------------------------------ 1080
170RII     ------------------------------------------------------------ 1080

expected   ACATAACCTTCTGATTTGCACAATAAATTACATATATTTAGCAGGATTTTTATTTGCCGT 1140
genomic    ------------------------------------------------------------ 1140
170FI      ACATAACCTTCTGATTTGC----------------------------------------- 1140
170FII     ACATAACCTTCTGATTT------------------------------------------- 1140
324RI      ACATAACCTTYTGATTTGCACAATAAATTACATATATTTAGCMGGATTTTTWTTTGCCST 1140
324RII     ACATAACCTTYTGATTTGCACAATAAATTACAWATATTTAGCMGGATTTTTATTTGCCST 1140
324FI      ------------------------------------------------------------ 1140
324FII     ------------------------------------------------------------ 1140
170RI      ------------------------------------------------------------ 1140
170RII     ------------------------------------------------------------ 1140

expected   GATATATAGGATAATTTAGTCTTTGGCATGTGGCATTATATTTATTTTGGTTTTTTTTTT 1200
genomic    ------------------------------------------------------------ 1200
170FI      ------------------------------------------------------------ 1200
170FII     ------------------------------------------------------------ 1200
324RI      GATATATAGGATAAWTTAGTYTTTGGCMTGKGGCMTTATATTTATTTTGGTTTTTTTTTT 1200
324RII     GATATATAGGATAAWTTAGTYTTTGGCMTGTGGCMTTATATTTATTTTGGTTTTTTTTTT 1200
324FI      ------------------------------------------------------------ 1200
324FII     ------------------------------------------------------------ 1200
170RI      ------------------------------------------------------------ 1200
170RII     ------------------------------------------------------------ 1200
```

```
expected   TAAACAGGTTATCAACGAAACTTCTCAGCATCACGATGACCTTGAAggaagcggagaggg 1260
genomic    ------------------------------------------------------------ 1260
170FI      ------------------------------------------------------------ 1260
170FII     ------------------------------------------------------------ 1260
324RI      TAAACAGGTTATCAACGAAACTTCTYAGCATCACGATGACCTTGAAGGAAGCGGAGAGGG 1260
324RII     TAAACAGGTTATCAACGAAACTTCTYAGCATCACGATGACCYTGAAGGAAGCGGAGAGGG 1260
324FI      ------------------------------------------------------------ 1260
324FII     ------------------------------------------------------------ 1260
170RI      ------------------------------------------------------------ 1260
170RII     ------------------------------------------------------------ 1260

expected   cagaggaagtctgctaacatgcggtgacgtcgaggagaatcctggacctATGGTGAGCAA 1320
genomic    ------------------------------------------------------------ 1320
170FI      ------------------------------------------------------------ 1320
170FII     ------------------------------------------------------------ 1320
324RI      CAGRGGAAGTCTGSTAACMTGCGGTGACGTCGAGGAGAATCCTGGACCTATGGTGAGCAA 1320
324RII     CAGRGGAAGTCTGSTAACMTGCGGTGACGTCGAGGAGAATCCTGGACCTATGGTGAGCAA 1320
324FI      ------------------------------------------------------------ 1320
324FII     ------------------------------------------------------------ 1320
170RI      ------------------------------------------------------------ 1320
170RII     ------------------------------------------------------------ 1320

expected   GGGCGAGGAGCTGATCAAGGAGAACATGCACATGAAGCTGTACATGGAAGGCACCGTGAA 1380
genomic    ------------------------------------------------------------ 1380
170FI      ------------------------------------------------------------ 1380
170FII     ------------------------------------------------------------ 1380
324RI      GGGCGAGGAGCTGATCAAGGAGAACATGCACATGAAGCTGTACATGGAAGGC-------- 1380
324RII     GGGCGAGGAGCTGATCAAGGAGAACATGCACATGAAGCTGTACATGGAAGGCACC----- 1380
324FI      ------------------------------------------------------------ 1380
324FII     ------------------------------------------------------------ 1380
170RI      ------------------------------------------------------------ 1380
170RII     ------------------------------------------------------------ 1380

expected   CAACCACCACTTCAAGTGCACCACCGAAGGGGAGGGCAAGCCCTACGAGGGCACCCAGAC 1440
genomic    ------------------------------------------------------------ 1440
170FI      ------------------------------------------------------------ 1440
170FII     ------------------------------------------------------------ 1440
324RI      ------------------------------------------------------------ 1440
324RII     ------------------------------------------------------------ 1440
324FI      -------------------------------GAGGGCAAGCCCTACGAGGGCACCCAGAC 1440
324FII     ---------------------------------GGGCAAGCCCTACGAGGGCACCCAGAC 1440
170RI      ------------------------------------------------------------ 1440
170RII     ------------------------------------------------------------ 1440

expected   CCAGAGGATTAAGGTGGTGGAGGGAGGCCCCCTGCCGTTCGCATTCGACATCCTGGCCAC 1500
```

```
genomic     ------------------------------------------------------------ 1500
170FI       ------------------------------------------------------------ 1500
170FII      ------------------------------------------------------------ 1500
324RI       ------------------------------------------------------------ 1500
324RII      ------------------------------------------------------------ 1500
324FI       CCAGAGGATTAAGGTGGTGGAGGGAGGCCCCCTGCCGTTCGCATTCGACATCCTGGCCAC 1500
324FII      CCAGAGGATTAAGGTGGTGGAGGGAGGCCCCCTGCCGTTCGCATTCGACATCCTGGCCAC 1500
170RI       ------------------------------------------------------------ 1500
170RII      ------------------------------------------------------------ 1500

expected    CTGCTTTATGTACGGGAGCAAGACCTTCATCAACCACACCCAGGGCATCCCCGATTTCTT 1560
genomic     ------------------------------------------------------------ 1560
170FI       ------------------------------------------------------------ 1560
170FII      ------------------------------------------------------------ 1560
324RI       ------------------------------------------------------------ 1560
324RII      ------------------------------------------------------------ 1560
324FI       CTGCTTTATGTACGGGAGCAAGACCTTCATCAACCACACCCAGGGCATCCCCGATTTCTT 1560
324FII      CTGCTTTATGTACGGGAGCAAGACCTTCATCAACCACACCCAGGGCATCCCCGATTTCTT 1560
170RI       ------------------------------------------------------------ 1560
170RII      ------------------------------------------------------------ 1560

expected    TAAGCAGTCCTTCCCTGAGGGCTTCACATGGGAGAGAGTCACCACATACGAAGACGGGGG 1620
genomic     ------------------------------------------------------------ 1620
170FI       ------------------------------------------------------------ 1620
170FII      ------------------------------------------------------------ 1620
324RI       ------------------------------------------------------------ 1620
324RII      ------------------------------------------------------------ 1620
324FI       TAAGCAGTCCTTCCCTGAGGGCTTCACATGGGAGAGAGTCACCACATACGAAGACGGGGG 1620
324FII      TAAGCAGTCCTTCCCTGAGGGCTTCACATGGGAGAGAGTCACCACATACGAAGACGGGGG 1620
170RI       ------------------------------------------------------------ 1620
170RII      ------------------------------------------------------------ 1620

expected    CGTGCTTACCGTTACCCAGGACACCAGCCTCCAGGACGGCTGCTTGATCTACAACGTCAA 1680
genomic     ------------------------------------------------------------ 1680
170FI       ------------------------------------------------------------ 1680
170FII      ------------------------------------------------------------ 1680
324RI       ------------------------------------------------------------ 1680
324RII      ------------------------------------------------------------ 1680
324FI       CGTGCTTACCGTTACCCAGGACACCAGCCTCCAGGACGGCTGCTTGATCTACAACGTCAA 1680
324FII      CGTGCTTACCGTTACCCAGGACACCAGCCTCCAGGACGGCTGCTTGATCTACAACGTCAA 1680
170RI       ------------------------------------------------------------ 1680
170RII      ------------------------------------------------------------ 1680

expected    GCTCAGAGGGGTGAACTTCCCATCCAACGGCCCTGTGATGCAGAAGAAAACACTCGGCTG 1740
genomic     ------------------------------------------------------------ 1740
```

```
170FI      ------------------------------------------------------------ 1740
170FII     ------------------------------------------------------------ 1740
324RI      ------------------------------------------------------------ 1740
324RII     ------------------------------------------------------------ 1740
324FI      GCTCAGAGGGGTGAACTTCCCATCCAACGGCCCTGTGATGCAGAAGAAAACACTCGGCTG 1740
324FII     GCTCAGAGGGGTGAACTTCCCATCCAACGGCCCTGTGATGCAGAAGAAAACACTCGGCTG 1740
170RI      ------------------------------------------------------------ 1740
170RII     ------------------------------------------------------------ 1740

expected   GGAGGCCACCACCGAGACCCTGTACCCCGCTGACGGCGGCCTGGAAGGCAGATGCGACAT 1800
genomic    ------------------------------------------------------------ 1800
170FI      ------------------------------------------------------------ 1800
170FII     ------------------------------------------------------------ 1800
324RI      ------------------------------------------------------------ 1800
324RII     ------------------------------------------------------------ 1800
324FI      GGAGGCCACCACCGAGACCCTGTACCCCGCTGACGGCGGCCTGGAAGGCAGATGCGACAT 1800
324FII     GGAGGCCACCACCGAGACCCTGTACCCCGCTGACGGCGGCCTGGAAGGCAGATGCGACAT 1800
170RI      ------------------------------------------------------------ 1800
170RII     ------------------------------------------------------------ 1800

expected   GGCCCTGAAGCTCGTGGGCGGGGGCCACCTGCACTGCAACCTGAAGACCACATACAGATC 1860
genomic    ------------------------------------------------------------ 1860
170FI      ------------------------------------------------------------ 1860
170FII     ------------------------------------------------------------ 1860
324RI      ------------------------------------------------------------ 1860
324RII     ------------------------------------------------------------ 1860
324FI      GGCCCTGAAGCTCGTGGGCGGGGGCCACCTGCACTGCAACCTGAAGACCACATACAGATC 1860
324FII     GGCCCTGAAGCTCGTGGGCGGGGGCCACCTGCACTGCAACCTGAAGACCACATACAGATC 1860
170RI      ------------------------------------------------------------ 1860
170RII     ------------------------------------------------------------ 1860

expected   CAAGAAACCCGCTAAGAACCTCAAGATGCCCGGCGTCTACTTTGTGGACCGCAGACTGGA 1920
genomic    ------------------------------------------------------------ 1920
170FI      ------------------------------------------------------------ 1920
170FII     ------------------------------------------------------------ 1920
324RI      ------------------------------------------------------------ 1920
324RII     ------------------------------------------------------------ 1920
324FI      CAAGAAACCCGCTAAGAACCTCAAGATGCCCGGCGTCTACTTTGTGGACCGCAGACTGGA 1920
324FII     CAAGAAACCCGCTAAGAACCTCAAGATGCCCGGCGTCTACTTTGTGGACCGCAGACTGGA 1920
170RI      ------------------------------------------------------------ 1920
170RII     ------------------------------------------------------------ 1920

expected   AAGAATCAAGGAGGCCGACAATGAGACCTACGTCGAGCAGCACGAGGTGGCTGTGGCCAG 1980
genomic    ------------------------------------------------------------ 1980
170FI      ------------------------------------------------------------ 1980
```

```
170FII      ------------------------------------------------------------ 1980
324RI       ------------------------------------------------------------ 1980
324RII      ------------------------------------------------------------ 1980
324FI       AAGAATCAAGGAGGCCGACAATGAGACCTACGTCGAGCAGCACGAGGTGGCTGTGGCCAG 1980
324FII      AAGAATCAAGGAGGCCGACAATGAGACCTACGTCGAGCAGCACGAGGTGGCTGTGGCCAG 1980
170RI       ------------------------------------------------------------ 1980
170RII      ------------------------------------------------------------ 1980

expected    ATACTGCGACCTCCCTAGCAAACTGGGGCACAAACTTAATGGCATGGACGAGCTGTACAA 2040
genomic     ------------------------------------------------------------ 2040
170FI       ------------------------------------------------------------ 2040
170FII      ------------------------------------------------------------ 2040
324RI       ------------------------------------------------------------ 2040
324RII      ------------------------------------------------------------ 2040
324FI       ATACTGCGACCTCCCTAGCAAACTGGGGCACAAACTTAATGGCATGGACGAGCTGTACAA 2040
324FII      ATACTGCGACCTCCCTAGCAAACTGGGGCACAAACTTAATGGCATGGACGAGCTGTACAA 2040
170RI       ------------------------------------------------------------ 2040
170RII      ------------------------------------------------------------ 2040

expected    GGACTATAAAGACCACGACGGAGACTACAAGGATCATGATATTGATTACAAAGACGATGA 2100
genomic     ------------------------------------------------------------ 2100
170FI       ------------------------------------------------------------ 2100
170FII      ------------------------------------------------------------ 2100
324RI       ------------------------------------------------------------ 2100
324RII      ------------------------------------------------------------ 2100
324FI       GGACTATAAAGACCACGACGGAGACTACAAGGATCATGATATTGATTACAAAGACGATGA 2100
324FII      GGACTATAAAGACCACGACGGAGACTACAAGGATCATGATATTGATTACAAAGACGATGA 2100
170RI       ----------GACCACGACGGAGACTACAAGGATCATGATATTGATTACAAAGACGATGA 2100
170RII      -------------------------------------------------AAAGACGATGA 2100

expected    CGATAAGTAATAGTGGAAGAGGGTCTTGTGTGTCTGCCCCTTCTAGTTTTCACTCATGCA 2160
genomic     ---------GTAGTGGAAGAGGGTCTTGTGTGTCTGCCCCTTCTAGTTTTCACTCATGCA 2160
170FI       ------------------------------------------------------------ 2160
170FII      ------------------------------------------------------------ 2160
324RI       ------------------------------------------------------------ 2160
324RII      ------------------------------------------------------------ 2160
324FI       CGATAAGTAATAGTGGAAGAGGGTCTTGTGTGTCTGCCCCTTCTAGTTTTCACTCATGCA 2160
324FII      CGATAAGTAATAGTGGAAGAGGGTCTTGTGTGTCTGCCCCTTCTAGTTTTCACTCATGCA 2160
170RI       CGATAAGTAATAGTGGAAGAGGGTCTTGTGTGTCTGCCCCTTCTAGTTTTCACTCATGCA 2160
170RII      CGATAAGTTATAGTGGAAGAGGGTCTTGTGTGTCTGCCCCTTCTAGTTTTCACTCATGCA 2160

expected    GAAGCAACATAACCTTCTGATTTGCACAATAAATTACATATATTTAGCAGGATTTTTATT 2220
genomic     GAAGCAACATAACCTTCTGATTTGCACAATAAATTACATATATTTAGCAGGATTTTTATT 2220
170FI       ------------------------------------------------------------ 2220
170FII      ------------------------------------------------------------ 2220
```

```
324RI       ------------------------------------------------------------ 2220
324RII      ------------------------------------------------------------ 2220
324FI       GAAGCAACATAACCTTCTGATTTGCACAATAAATTACATATATTTAGCAGGATTTTTATT 2220
324FII      GAAGCAACATAACCTTCTGATTTGCACAATAAATTACATATATTTAGCAGGATTTTTATT 2220
170RI       GAAGCAACATAACCTTCTGATTTGCACAATAAATTACATATATTTAGCAGGATTTTTATT 2220
170RII      GAAGCAACATAACCTTCTGATTTGCACAATAAATTACATATATTTAGCAGGATTTTTATT 2220

expected    TGCCGTGATATATAGGATAATTTAGTCTTTGGCATGTGGCATTATATTTATTTTGGTTTT 2280
genomic     TGCCGTGATATATAGGATAATTTAGTCTTTGGCATGTGGCATTATATTTATTTTGGTTTT 2280
170FI       ------------------------------------------------------------ 2280
170FII      ------------------------------------------------------------ 2280
324RI       ------------------------------------------------------------ 2280
324RII      ------------------------------------------------------------ 2280
324FI       TGCCGTGATATATAGGATAATTTAGTCTTTGGCATGTGGCATTATATTTATTTTGG-TTT 2280
324FII      TGCCGTGAtATATAGGATAATTTAGTCTTTGGCATGTGGCATTATATTTATTTTGG-TTT 2280
170RI       TGCCGTGATATATAGGATAATTTAGTCTTTGGCATGTGGCATTATATTTATTTTGGTTTT 2280
170RII      TGCCGTGATATATAGGATAATTTAGTCTTTGGCATGTGGCATTATATTTATTTTGGTTTT 2280

expected    TTTTTTTAAACAGGTTATCAACGAAACTTCTCAGCATCACGATGACCTTGAATAAAAATT 2340
genomic     TTTTTTTAAACAGGTTATCAACGAAACTTCTCAGCATCACGATGACCTTGAATAAAAATT 2340
170FI       ------------------------------------------------------------ 2340
170FII      ------------------------------------------------------------ 2340
324RI       ------------------------------------------------------------ 2340
324RII      ------------------------------------------------------------ 2340
324FI       TTTTTTTAAACAGGTTATCAACGAAACTTCTCAGCATCACGATGACCTTGAATAAAAATT 2340
324FII      TTTTTTTAAACAGGTTATCAACGAAACTTCTCAGCATCACGATGACCTTGAATAAAAATT 2340
170RI       TTTTTTTAAACAGGTTATCAACGAAACTTCTCAGCATCACGATGACCTTGAATAAAAATT 2340
170RII      TTTTTTTAAACAGGTTATCAACGAAACTTCTCAGCATCACGATGACCTTGAATAAAAATT 2340

expected    GCACACACTCAGTGCAGCAATATATTACCAGCAAGAATAAAAAAGAAATCCATATCTTAA 2400
genomic     GCACACACTCAGTGCAGCAATATATTACCAGCAAGAATAAAAAAGAAATCCATATCTTAA 2400
170FI       ------------------------------------------------------------ 2400
170FII      ------------------------------------------------------------ 2400
324RI       ------------------------------------------------------------ 2400
324RII      ------------------------------------------------------------ 2400
324FI       GCAC-------------------------------------------------------- 2400
324FII      GCACACACTCAGTGCAGCAATATATTACCAGCAAGA--TAAAAAGAAATCCATATC-TAA 2400
170RI       GCACACACTCAGTGCAGCAATATATTACCAGCAAGAATAAAAAAGAAATCCATATCTTAA 2400
170RII      GCACACACTCAGTGCAGCAATATATTACCAGCAAGAATAAAAAAGAAATCCATATCTTAA 2400

expected    AGAAACAGCTTTCAAGTGCCTTTCTGCAGTTTTTCAGGAGCGCAAGATAGATTTGGAATA 2460
genomic     AGAAACAGCTTTCAAGTGCCTTTCTGCAGTTTTTCAGGAGCGCAAGATAGATTTGGAATA 2460
170FI       ------------------------------------------------------------ 2460
170FII      ------------------------------------------------------------ 2460
324RI       ------------------------------------------------------------ 2460
```

```
324RII      ------------------------------------------------------------ 2460
324FI       ------------------------------------------------------------ 2460
324FII      AGAAACAGCTTTC-AGTGCCTTTCTGCAGTTTT--------------------------- 2460
170RI       AGAAACAGCTTTCAAGTGCCTTTCTGCAGTTTTTCAGGAGCGCAAGATAGATTTGGAATA 2460
170RII      AGAAACAGCTTTCAAGTGCCTTTCTGCAGTTTTTCAGGAGCGCAAGATAGATTTGGAATA 2460

expected    GGAATAAGCTCTAGTTCTTAACAACCGACACTCCTACAAGATTTAGAAAAAAGTTTACAA 2520
genomic     GGAATAAGCTCTAGTTCTTAACAACCGACACTCCTACAAGATTTAGAAAAAAGTTTACAA 2520
170FI       ------------------------------------------------------------ 2520
170FII      ------------------------------------------------------------ 2520
324RI       ------------------------------------------------------------ 2520
324RII      ------------------------------------------------------------ 2520
324FI       ------------------------------------------------------------ 2520
324FII      ------------------------------------------------------------ 2520
170RI       GGAATAAGCTCTAGTTCTTAACAACCGACACTCCTACAAGATTTAGAAAAAAGTTTACAA 2520
170RII      GGAATAAGCTCTAGTTCTTAACAACCGACACTCCTACAAGATTTAGAAAAAAGTTTACAA 2520

expected    CATAATCTAGTTTACAGAAAAATCTTGTGCTAGAATACTTTTTAAAAGGTATTTTGAATA 2580
genomic     CATAATCTAGTTTACAGAAAAATCTTGTGCTAGAATACTTTTTAAAAGGTATTTTGAATA 2580
170FI       ------------------------------------------------------------ 2580
170FII      ------------------------------------------------------------ 2580
324RI       ------------------------------------------------------------ 2580
324RII      ------------------------------------------------------------ 2580
324FI       ------------------------------------------------------------ 2580
324FII      ------------------------------------------------------------ 2580
170RI       CATAATCTAGTTTACAGAAAAATCTTGTGCTAGAATACTTTTTAAAAGGTATTTTGAATA 2580
170RII      CATAATCTAGTTTACAGAAAAATCTTGTGCTAGAATACTTTTTAAAAGGTATTTTGAATA 2580

expected    CCATTAAAACTGCTTTTTTTTTTCCAGCAAGTATCCAACCAACTTGGTTCTGCTTCAATA 2640
genomic     CCATTAAAACTGCTTTTTTTTTTCCAGCAAGTATCCAACCAACTTGGTTCTGCTTCAATA 2640
170FI       ------------------------------------------------------------ 2640
170FII      ------------------------------------------------------------ 2640
324RI       ------------------------------------------------------------ 2640
324RII      ------------------------------------------------------------ 2640
324FI       ------------------------------------------------------------ 2640
324FII      ------------------------------------------------------------ 2640
170RI       CCATTAAAACTGCTTTTTTTTTTCCAGCAAGTATCCAACCAACTTGGTTCTGCTTCAATA 2640
170RII      CCATTAAAACTGCTTTTTTTTTTCCAGCAAGTATCCAACCAACTTGGTTCTGCTTCAATA 2640

expected    AATCTTTGGAAAAACTCTTTTGTTGTGTTATTTATTGGATAATATCTAAACAATTCTCTA 2700
genomic     AATCTTTGGAAAAACTCTTTTGTTGTGTTATTTATTGGATAATATCTAAACAATTCTCTA 2700
170FI       ------------------------------------------------------------ 2700
170FII      ------------------------------------------------------------ 2700
324RI       ------------------------------------------------------------ 2700
324RII      ------------------------------------------------------------ 2700
```

```
324FI       ------------------------------------------------------------ 2700
324FII      ------------------------------------------------------------ 2700
170RI       AATCTTTGGAAAAACTCTTTTGTTGTGTTATTTATTGGATAATATCTAAACAATTCTCTA 2700
170RII      AATCTTTGGAAAAACTCTTTTGTTGTGTTATTTATTGGATAATATCTAAACAATTCTCTA 2700

expected    CTTGGTCCTATTAGTTAATTTGTCATTACAATCATGTAAGTTGATAAATTCAGGTTATTT 2760
genomic     CTTGGTCCTATTAGTTAATTTGTCATTACAATCATGTAAGTTGATAAATTCAGGTTATTT 2760
170FI       ------------------------------------------------------------ 2760
170FII      ------------------------------------------------------------ 2760
324RI       ------------------------------------------------------------ 2760
324RII      ------------------------------------------------------------ 2760
324FI       ------------------------------------------------------------ 2760
324FII      ------------------------------------------------------------ 2760
170RI       CTTGGTCCTATTAGTTAATTTGTCATTACAATCATGTAAGTTGATAAATTCAGGTTATTT 2760
170RII      CTTGGTCCTATTAGTTAATTTGTCATTACAATCATGTAAGTTGATAAATTCAGGTTATTT 2760

expected    ATGCTTGAGATGTAGTTCTTAATTTTGTCATTTTTGATAGACCTCACTTCTTTTTATTAT 2820
genomic     ATGCTTGAGATGTAGTTCTTAATTTTGTCATTTTTGATAGACCTCACTTCTTTTTATTAT 2820
170FI       ------------------------------------------------------------ 2820
170FII      ------------------------------------------------------------ 2820
324RI       ------------------------------------------------------------ 2820
324RII      ------------------------------------------------------------ 2820
324FI       ------------------------------------------------------------ 2820
324FII      ------------------------------------------------------------ 2820
170RI       ATGCTTGAGATGTAGTTCTTAATTTTGTCATTTTTGATAGACCTCACTTCTTTTTATTAT 2820
170RII      ATGCTTGAGATGTAGTTCTTAATTTTGTCATTTTTGATAGACCTCACTTCTTTTTATTAT 2820

expected    TACTTAAAAACATTTACAAATAGGTGGTGTCGAATAAAATAACTTGTACCAAAATGAAGA 2880
genomic     TACTTAAAAACATTTACAAATAGGTGGTGTCGAATAAAATAACTTGTACCAAAATGAAGA 2880
170FI       ------------------------------------------------------------ 2880
170FII      ------------------------------------------------------------ 2880
324RI       ------------------------------------------------------------ 2880
324RII      ------------------------------------------------------------ 2880
324FI       ------------------------------------------------------------ 2880
324FII      ------------------------------------------------------------ 2880
170RI       TACTTAAAAACATTTACAAATAGGTGGTGTCGAATAAAATAACTTGTACCAAAATGAAGA 2880
170RII      TACTTAAAAACATTTACAAATAGGTGGTGTCGAATAAAATAACTTGTACCAAAATGAAGA 2880

expected    TAGGTCTCTCTAAAATGAGCTCAGGTCTGTGATTTTAATCATAACAAACAGACTTCCTAA 2940
genomic     TAGGTCTCTCTAAAATGAGCTCAGGTCTGTGATTTTAATCATAACAAACAGACTTCCTAA 2940
170FI       ------------------------------------------------------------ 2940
170FII      ------------------------------------------------------------ 2940
324RI       ------------------------------------------------------------ 2940
324RII      ------------------------------------------------------------ 2940
324FI       ------------------------------------------------------------ 2940
```

```
324FII      ------------------------------------------------------------ 2940
170RI       TAGGTCTCTCTAAAATGAGCTCAGGTCTGTGATTTTAATCATAACAAACAGACTTCCTAA 2940
170RII      TAGGTCTCTCTAAAATGAGCTCAGGTCTGTGATTTTAATCATAACAAACAGACTTCCTAA 2940

expected    AATTAAAAAATAAAAACTTTTTTTAGTACATATAGCATATGAGTAACACAAATTCCACTT 3000
genomic     AATTAAAAAATAAAAACTTTTTTTAGTACATATAGCATATGAGTAACACAAATTCCACTT 3000
170FI       ------------------------------------------------------------ 3000
170FII      ------------------------------------------------------------ 3000
324RI       ------------------------------------------------------------ 3000
324RII      ------------------------------------------------------------ 3000
324FI       ------------------------------------------------------------ 3000
324FII      ------------------------------------------------------------ 3000
170RI       AATTAAAAAATAAAAACTTTTTTTAGTACATATAGCATATGAGTAACACA---------- 3000
170RII      AATTAAAAAATAAAAACTTTTTTTAGTACATATAGCATATGAGTAACACAAA-------- 3000

expected    TGGGAGTTAAAGCATAGGAAGTTGCCAAGATATAGGGGCTTATCTTCCGCTAGCAAGATG 3060
genomic     TGGGAGT----------------------------------------------------- 3060
170FI       ------------------------------------------------------------ 3060
170FII      ------------------------------------------------------------ 3060
324RI       ------------------------------------------------------------ 3060
324RII      ------------------------------------------------------------ 3060
324FI       ------------------------------------------------------------ 3060
324FII      ------------------------------------------------------------ 3060
170RI       ------------------------------------------------------------ 3060
170RII      ------------------------------------------------------------ 3060

expected    CAGAGAAATG 3070
genomic     ---------- 3070
170FI       ---------- 3070
170FII      ---------- 3070
324RI       ---------- 3070
324RII      ---------- 3070
324FI       ---------- 3070
324FII      ---------- 3070
170RI       ---------- 3070
170RII      ---------- 3070
```

Figure S2. The DNA template for knock-in into *Vimentin* gene. Partial vKiT plasmid sequence which contains the template for the knock-in. The sequence recognized by Cas9/gRNA complex was recoded are shown in red text. Blue text are the PAM sequences.

VIM gRNA target site – 5’HA – VIM missing part - T2A - mCardinal –3xFLAG- 3’HA - VIM gRNA target site

**GATCCTTATATTGCCTGTAGTGGAAGAAAAAACTCGTTTTTACTCATTTTTGGCCTGTTTGTTTATTTAGAAACTAATCT
GGATTCACTCCCTCTGGTTGATACCCACTCAAAAAGGACACTTCTGATTAAGACGGTTGAAACTAGAGATGGACAGGTTG
GTATCTTTTAAGGAAAAAATAGGGTAATCTCAGACAGGAGTTGATATATTTTAAAATCAGTGAATCTGAATCTCAGATAC
AGCTGGCTAATTTGAGAGGTTCAGGTTTCATTCATGCCTACTAAAAAAAGAATAGGCTTCTTCTTCCAGCAGTACACACA**

GCCAACTAATTATTTGGCTCCTGGATGTGAAGTTGAGATAGCAGTCTTCCTGTGCTCCAGAATTAGTGATTTGCTTTGGT
GCTTAATTTGAAGTGGGAGTAAGCTTCCTTAAACCACTTCCTAAAGCAGCTACATGAAACAGCTTCACTAGACTACCTCA
ATATGAGGAATGTTTTGATCCTGGACATATGGTGTCTTCCTACCTCCATACTTTATAGATTCCTAAACCCATCTATATAA
TACAAGCATGTGCCATACGATCATTTAGTTTCTTATTACCTCCCTATGCCAGGAAAGAAATAGTTGCAATTTATTGTAGT
CATCATGAAATCTTCCCTTGCACATAAATTTAAAATGTACCTGCTGCACATTTTAATATGTCTTAATTGCTTTTAAACTT
GGCTGTATTGTGTACAACTATTATACCATCTTTTATAAACACAGTTTTTTAAGAAATTTCTTTTTGTAAGTTACAACATT
CCACTGGA*tccttat***AGGACATATAATAC**AAGAGGGTCTTGTGTGTCTGCCCCTTCTAGTTTTCACTCATGCAGAAGCAA
CATAACCTTCTGATTTGCACAATAAATTACATATATTTAGCAGGATTTTTATTTGCCGTGATATATAGGATAATTTAGTC
TTTGGCATGTGGCATTATATTTATTTTGGTTTTTTTTTTTAAACAGGTTATCAACGAAACTTCTCAGCATCACGATGACC
TTGAAggaagcggagagggcagaggaagtctgctaacatgcggtgacgtcgaggagaatcctggacctATGGTGAGCAAGGGCGAGGAGCTGATCAA
GGAGAACATGCACATGAAGCTGTACATGGAAGGCACCGTGAACAACCACCACTTCAAGTGCACCACCGAAGGGGAGGGCAAGCC
CTACGAGGGCACCCAGACCCAGAGGATTAAGGTGGTGGAGGGAGGCCCCCTGCCGTTCGCATTCGACATCCTGGCCACCTGCTTT
ATGTACGGGAGCAAGACCTTCATCAACCACACCCAGGGCATCCCCGATTTCTTTAAGCAGTCCTTCCCTGAGGGCTTCACATGGGA
GAGAGTCACCACATACGAAGACGGGGGCGTGCTTACCGTTACCCAGGACACCAGCCTCCAGGACGGCTGCTTGATCTACAACGTC
AAGCTCAGAGGGGTGAACTTCCCATCCAACGGCCCTGTGATGCAGAAGAAAACACTCGGCTGGGAGGCCACCACCGAGACCCTGT
ACCCCGCTGACGGCGGCCTGGAAGGCAGATGCGACATGGCCCTGAAGCTCGTGGGCGGGGGCCACCTGCACTGCAACCTGAAGA
CCACATACAGATCCAAGAAACCCGCTAAGAACCTCAAGATGCCCGGCGTCTACTTTGTGGACCGCAGACTGGAAAGAATCAAGGA
GGCCGACAATGAGACCTACGTCGAGCAGCACGAGGTGGCTGTGGCCAGATACTGCGACCTCCCTAGCAAACTGGGGCACAAACTT
AATGGCATGGACGAGCTGTACAAGGACTA**TAAA**GACCACGACGGAGACTACAAGGATCATGATATTGATTACAAAGACG
ATGACGATAAGTAATAGTGGAAGAGGGTCTTGTGTGTCTGCCCCTTCTAGTTTTCACTCATGCAGAAGCAACATAACCTTCTGATT
TGCACAATAAATTACATATATTTAGCAGGATTTTTATTTGCCGTGATATATAGGATAATTTAGTCTTTGGCATGTGGCATTATATTTA
TTTTGGTTTTTTTTTTTAAACAGGTTATCAACGAAACTTCTCAGCATCACGATGACCTTGAATAAAAATTGCACACACTCAGTGCAGC
AATATATTACCAGCAAGAATAAAAAAGAAATCCATATCTTAAAGAAACAGCTTTCAAGTGCCTTTCTGCAGTTTTTCAGGAGCGCA
AGATAGATTTGGAATAGGAATAAGCTCTAGTTCTTAACAACCGACACTCCTACAAGATTTAGAAAAAAGTTTACAACATAATCTAG
TTTACAGAAAAATCTTGTGCTAGAATACTTTTTAAAAGGTATTTTGAATACCATTAAAACTGCTTTTTTTTTTCCAGCAAGTATCCAAC
CAACTTGGTTCTGCTTCAATAAATCTTTGGAAAAACTCTTTTGTTGTGTTATTTATTGGATAATATCTAAACAATTCTCTACTTGGTCC
TATTAGTTAATTTGTCATTACAATCATGTAAGTTGATAAATTCAGGTTATTTATGCTTGAGATGTAGTTCTTAATTTTGTCATTTTTGA
TAGACCTCACTTCTTTTTATTATTACTTAAAAACATTTACAAATAGGTGGTGTCGAATAAAATAACTTGTACCAAAATGAAGATAGG
TCTCTCTAAAATGAGCTCAGGTCTGTCAC**TACAGGCAATATAAGGATC**

Figure S3. Confocal imaging of HEK293 cells transfected with re-cloned *VIM-T2A-mCardinal* **(A-C)** and fusion VIM-mCardinal **(D-F).** Cellular distribution of mCardinal in *VIM-T2A-mCardinal* expressing cells shows its strong fluorescence in single dot-like area **(B)**, as well as it is weakly diffused in the cytoplasm. VIM-mCardinal fusion protein aggregated in the cytoplasm **(E)**. **A)** merged, **B)** mCardinal, **C)** transmission light, **D)** merged, **E)** mCardinal, **F)** transmission light. The scale bar is 10 μm.

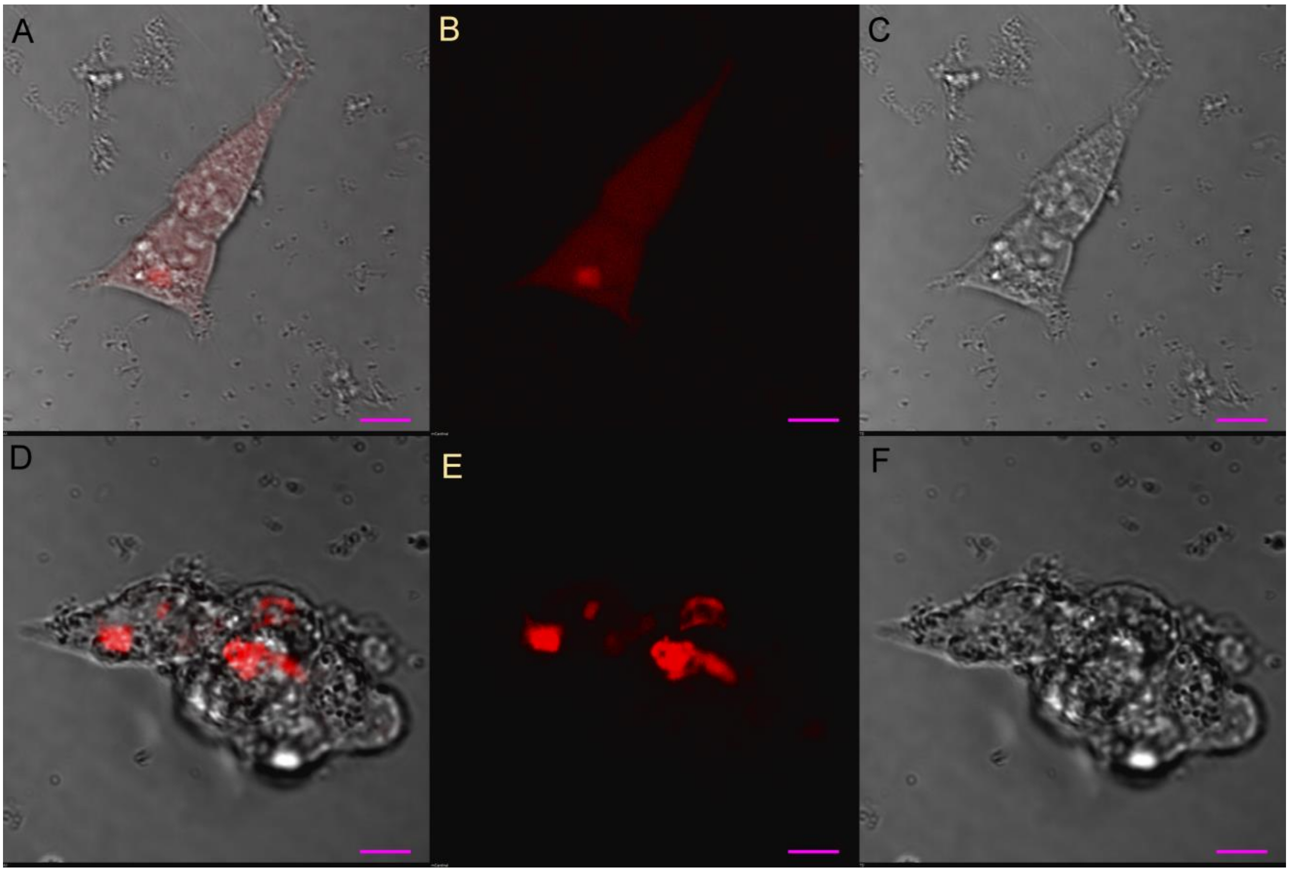

Figure S4. Sorting of VRCs by flow cytometry. **A)** P1 acquisition gate was set on FSC and SSC detectors. **B)** the cells showing red fluorescence were selected by P2 or P3 gate, then collected into the tubes and named as dim-VRCs or bright-VRCs respectively. Representative dot-plot has been shown.

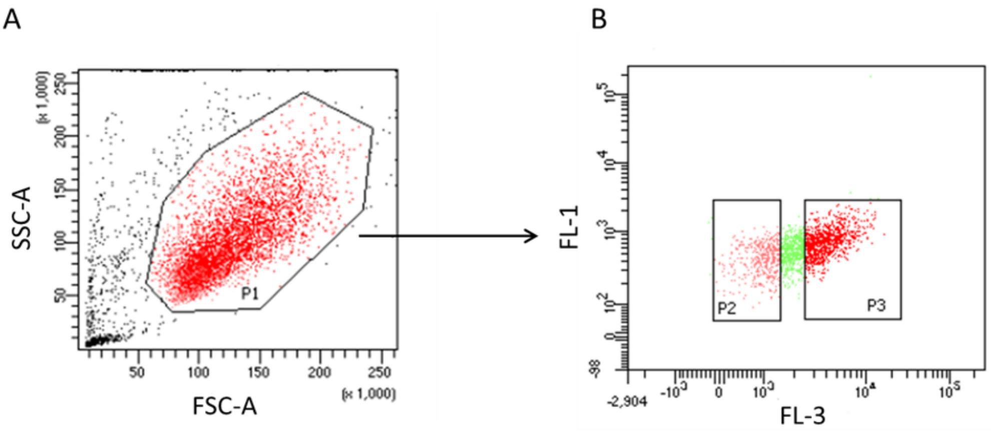

Figure S5. Expression of *VIM*, *mCardinal* and *CDH1* in dim-VRCs and bright-VRCs by qPCR. Mean 2^(−ΔCT) ± SEM is shown, the reference gene was *GAPDH*. The graph shows the representative result of the measurement which was done in triplicate.

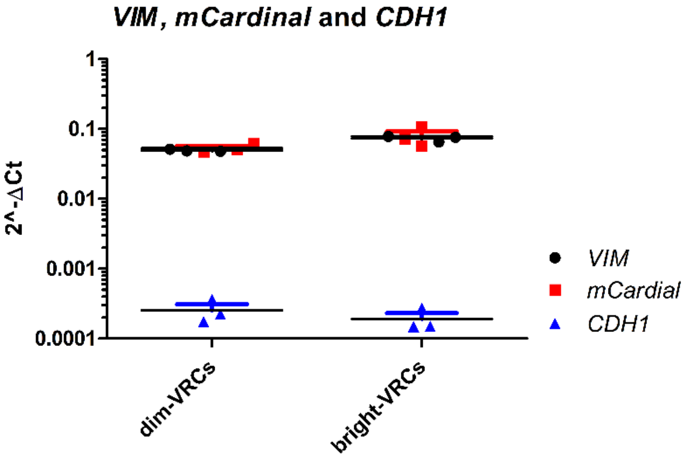

Figure S6. EMT markers in VRC and in H2170 parental cells assessed by qPCR. Relative quantification (RQ). Expression of *CDH1*, *VIM*, *SNAI1*, *ZEB1*, *ZEB2*, *TWIST1* and *TWIST2* in VRCs and in parental H2170 cell line has been shown as mean 2^(−ΔΔCT) ± SEM, with the *GAPDH* reference. The graph shows the representative result of the measurement which was done in triplicate.

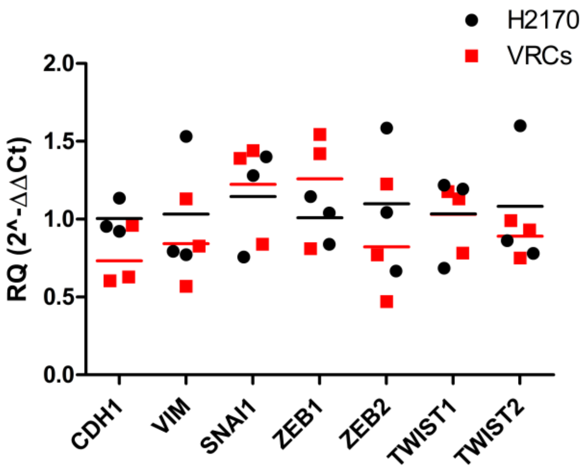

Table 1. Oligonucleotides.

	Name of the oligonucleotide	Sequence
1	gRNA-F	CACCGGATCCTTATATTGCCTGTAG
2	gRNA-R	AAACCTACAGGCAATATAAGGATCC
3	KI-F	ATGTCCACCAGGTCCGTGTC
4	KI-R	CAGACACACAAGACCCTCTTCCAC
5	BCB-F	AGGGTCTTGTGTGTCTGTCTCAGATCTCGAGCTCAAGCTTC
6	BCB-R	ACGGACCTGGTGGACATGTACGGAGACGGGATCTGACG
7	SDM-F	AGGAGAATGGCGGAGGGATGGTGAGCAAGGGCGAGG
8	SDM-R	CCCTCCGCCATTCTCCTCGACGTCACCGC
9	170	TCGAACCCAAGTACACTCTTGC
10	249	ATCCTTGTAGTCTCCGTCGTGGTC
11	GAPDH-F	CTCTGCTCCTCCTGTTCGAC
12	GAPDH-R	GCCCAATACGACCAAATCC
13	VIM-F	AGTCCACTGAGTACCGGAGAC
14	VIM-R	CATTTCACGCATCTGGCGTTC
15	mCard-F	TGATCAAGGAGAACATGCACATGAAGC
16	mCard-R	CCTTAATCCTCTGGGTCTGGGTG
17	Cdh1-F	CGAGAGCTACACGTTCACGG
18	Cdh1-R	GGGTGTCGAGGGAAAAATAGG
19	Mir145-F	AAGATCTCTACAGATGGGGCTGGATGC
20	Mir145-R	AAAGCTTCAAGAGTACGGCAGTGCTGA
21	Mir-200b-F	AAGATCTGGATTAGGACGCTCAGGTGTC
22	Mir-200b-R	AAAGCTTTGGAGTAGGAGCTCCGGATGTG
23	Mir-200c-F	AAGATCTAGGGTGGGTAAATCGGTGTG
24	Mir-200c-R	AAAGCTTACCTGAGGCGATGGATGTTG
25	Mir-205-F	AAGATCTCCTCCTTGGAGGATGTGA
26	Mir-205-R	AAAGCTTACGCACACTCCAGATGTCTC
27	ZEB1F	ACTGTGGTAGAAACAAATTCAGATTCAGATGATG
28	ZEB1R	GCCCTTCCTTTCCTGTGTCATCC
29	ZEB2F	CTCTGTAGATGGTCCAGTGAAGAATGC
30	ZEB2R	GTCACTGCGCTGAAGGTACTCC
31	SNAI1F	TCGGAAGCCTAACTACAGCGA
32	SNAI1R	AGATGAGCATTGGCAGCGAG
33	TWIST1F	GCCGGAGACCTAGATGTCATT
34	TWIST1R	TTTTAAAAGTGCGCCCCACG
35	TWIST2F	CGCCAGGGCTGTCCGTC
36	TWIST2R	GTCACTGCTGTCCCTTCTCTCG
